# Hydrophobic DES Based on Menthol and Natural Organic Acids for Use in Antifouling Marine Coatings

**DOI:** 10.1021/acssuschemeng.3c01120

**Published:** 2023-06-15

**Authors:** Sara Valente, Filipe Oliveira, Inês João Ferreira, Alexandre Paiva, Rita G. Sobral, Mário S. Diniz, Susana P. Gaudêncio, Ana Rita Cruz Duarte

**Affiliations:** † LAQV-REQUIMTE, Chemistry Department, NOVA School of Science and Technology, 2829-516 Caparica, Portugal; ‡ Associate Laboratory i4HB—Institute for Health and Bioeconomy, NOVA School of Science and Technology, 2829-516 Caparica, Portugal; § UCIBIO, Chemistry and Life Sciences Departments, NOVA School of Science and Technology, 2829-516 Caparica, Portugal

**Keywords:** hydrophobic deep eutectic systems (HDES), natural products, antibiofilm, antifouling, biocide-free, eco-friendly, non-toxic, marine fouling control, coatings and paints

## Abstract

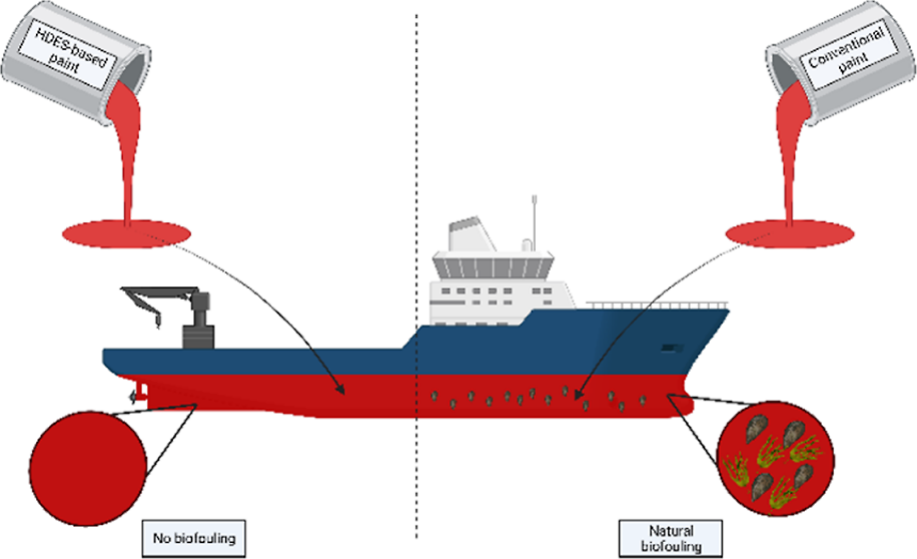

Marine biofouling negatively impacts industries with off-shore infrastructures, such as naval, oil, and aquaculture. To date, there are no ideal sustainable, economic, and environmentally benign solutions to deal with this phenomenon. The advances achieved in green solvents, as well as its application in different industries, such as pharmaceutical and biotechnology, have promoted the emergence of deep eutectic systems (DES). These eutectic systems have applications in various fields and can be revolutionary in the marine-based industrial sector. In this study, the main objective was to investigate the potential use of hydrophobic DES (HDES) based on menthol and natural organic acids for their use as marine antifouling coatings. Our strategy encompassed the physicochemical characterization of different formulations, which allowed us to identify the most appropriate molar ratio and intermolecular interactions for HDES formations. The miscibility of the resulting HDES with the marine coating has been evaluated and proven to be successful. The Men/OL (1:1) system proved to be the most promising in terms of cost-production and thus was the one used in subsequent antifouling tests. The cytotoxicity of this HDES was evaluated using an in vitro cell model (HaCat cells) showing no significant toxicity. Furthermore, the application of this system incorporated into coatings that are used in marine structures was also studied using marine species (*Mytilus edulis* mussels and *Patella vulgata* limpets) to evaluate both their antifouling and ecotoxicity effects. HDES Men/OL (1:1) incorporated in marine coatings was promising in reducing marine macrofouling and also proved to be effective at the level of microfouling without viability impairment of the tested marine species. It was revealed to be more efficient than using copper oxide, metallic copper, or ivermectin as antifouling agents. Biochemical assays performed on marine species showed that this HDES does not induce oxidative stress in the tested species. These results are a strong indication of the potential of this HDES to be sustainable and efficiently used in marine fouling control technologies.

## Abstract 2

Marine biofouling negatively impacts submerged infrastructures, and to date, there are no ideal sustainable, economic, and environmentally benign solutions to deal with this phenomenon. The results described herein provide compelling evidence that the use of deep eutectic systems may pave the way for alternative antifouling additive agents for marine coatings. The work that has been reported here gives a clear indication of the potential of HDES as efficient and sustainable marine fouling control methods.

## 1 Introduction

Marine biofouling is defined as unwanted colonization on the surface of submerged structures by micro (bacteria and diatoms) and macroorganisms (barnacles, mussels, polychaete worms, bryozoans, seaweeds, etc.).^1,2^ This phenomenon is one of the most relevant problems that marine offshore infrastructures and industries currently face since itthis is a dynamic process that begins immediately after submersion and takes hours to months for its development.^3,4^ It begins with the formation of a film layer by microorganisms on the surface of the substrate composed of organic materials (proteins, polysaccharides, and proteoglycans).^2^ Both bacteria and diatoms secrete extracellular polymeric substances that lead to biofilm formation, which leads to irreversible adhesion and stronger fixation. This biofilm stimulates macrofouling larva or spore adherence and the attachment of invertebrates and algae to submerged marine surfaces, evolving into a complex biological community.^2^ For that reason, inhibiting bacterial biofilm formation is of utmost importance to prevent biofouling formation.^4−6^

Marine biofouling leads to negative impacts on ships and offshore infrastructure, namely the amplification of surface roughness, fuel consumption, and biocorrosion.^7^

The increase in the ship’s drag resistance and weight induced by the accumulation of marine organisms on the ship’s hull can lead up to a 40% increase of fuel consumption, reduced manoeuvrability, a significant increase of CO_2_ and SO_2_ emissions, and increased transportation costs.^4,7^ Fouling removal operations therefore need to be frequent and long, thus becoming more expensive. Moreover, these cleaning processes generate numerous toxic substances that are often released into the ocean.^2^ The spread of foreign invasive species is also a consequence of this phenomenon, which leads to environmental imbalances.^1^

Currently, coatings containing antifouling compounds are recognized as the most regularly used strategy against marine biofouling.^1^ It is estimated that this method prevents the marine industry from spending about 60 billion euros per year on fuel and leads to a reduction in CO_2_ and SO_2_ emissions of 384 million and 3.6 million tons per year, respectively.^8^ In the past, a variety of toxic materials, including lead, mercury, and arsenic, have been used to control fouling organisms. In the 1960s, organotin compounds such as tributyltin (TBT) were introduced and proved to be the most effective antifouling agents. However, these were among the most toxic biocides, as they acted on both target and non-target marine organisms and were not readily degraded in the natural environment. These harmful effects led the International Maritime Organization to ban the application of these organotin products since Sept 2008.^9^

After the TBT ban, copper is currently the most used. The increase in the number of vessels that adopted antifouling coatings with copper as the main biocide replacing coatings composed of TBT usually in the form of cuprous oxide (Cu_2_O), metallic copper (Cu), or copper thiocyanate (CuCHNS) led to high levels of these substances in areas of intense marine operation.^10,11^ Environmental concerns about the effects of copper on the marine environment, together with its increasing market price, have promoted the discovery and development of new compounds with biocidal properties.^12^ This is the case, for example, of ivermectin, a dihydro derivative of avermectin, a macrocyclic lactone isolated from the actinomycete species *Streptomyces avermitilis*, which is commonly used in the treatment of parasitic worms and as an insecticide.^4^

According to Devashree et al.,^13^ “the global antifouling paints and coatings market was valued at $5.91 billion in 2021 and is projected to reach $13.18 billion by 2031, growing at a CAGR of 8.48% from 2022 to 2031”.

An emerging concept in research due to their unique and attractive properties is deep eutectic systems (DES).^14^ Introduced by Abbott et al. in 2014, DES are a class of green solvents that are potential alternatives to common organic solvents.^15,16^ DES are usually defined as a mixture of two or more compounds, a hydrogen bond acceptor (HBA) and a hydrogen bond donor (HBD), which in a specific molar ratio presents a significant decrease in the melting point of the system when compared to their individual compounds.^17^ This decrease in melting point is mainly attributed to the establishment of hydrogen bond interactions between the compounds and also to electrostatic interactions and van der Waals forces that play an important role.^18^

DES are highly flexible and versatile systems for various applications, possibly accounting for 10^6^ different combinations.^19^ Another interesting feature is that biological and physicochemical properties of DES can be adjusted by the nature of their components, along with an adequate selection of molar ratio and temperature.^18^ In addition to these characteristics, DES have other attractive properties, such as reduced preparation costs, no need for post-synthesis purification and environmental disposal, non-flammability, a wide polarity range, low volatility, chemical and thermal stability, water compatibility, biodegradability, and low toxicity profiles, which are attributes that enhance DES.^18^

More recently, in 2015, van Osch and his collaborators introduced the concept of hydrophobic DES (HDES).^20^ HDES are synthesized using poorly water-soluble components and can be obtained by combining choline chloride with phenolic compounds, menthol with carboxylic acids, and exclusively from carboxylic acids.^21^

With the focus on mitigating marine biofouling, in the present work, a literature review of molecules with antifouling activity was performed to select the most promising HDES compatible molecules, as well as an analysis of the HDES already described in the literature. In this sense, the selected commercial molecules were natural organic acids (namely, oleic acid and 3-hydroxybutyric acid) and terpenes (specifically, menthol), with demonstrated relevant scientific evidence that promoted antibiofouling activity against several marine species. The present study demonstrates the possibility of enhancing biocide-free marine coatings by exploring the antifouling activity of HDES. In this way, sustainable marine antifouling coatings can be developed based on eutectic mixtures prepared from natural resources in a cost-effective manner while minimizing the biofouling problem. To the best of our knowledge, HDES containing these antifouling molecules have never been reported in the literature.

Although the components that form the eutectic systems are derived from biomaterials, it is necessary to consider the synergistic effect of the combination of these compounds on HDES. Thus, the estimation of the sustainable potential of eutectic systems should be proactively evaluated before their large-scale use.^22^

## 2 Materials and Methods

### 2.1 HDES Preparation

A revision of the literature, using the Web of Science, was carried out to select natural commercial molecules with antifouling activity that were the most appropriate for synthesizing HDES. The selected molecules were natural organic acids (oleic acid and 3-hydroxybutyric acid) and terpenes (menthol). DES were prepared by mixing dl-menthol (Men) (W266507, Sigma-Aldrich, St. Louis, MO, USA) with oleic acid (OL) (W281506, Sigma-Aldrich, St. Louis, MO, USA) and with 3-hydroxybutyric acid (HB) (166898, Sigma-Aldrich, St. Louis, MO, USA) in different molar ratios. The systems were mixed under constant stirring at 30 °C. After 30 min, a clear liquid was obtained, and the HDES were left to cool at room temperature (RT). The Men/HB (2:1), Men/HB (3:1), and Men/OL (1:1) systems were prepared for this work.

### 2.2 Miscibility of the Developed Hydrophobic Eutectic Systems in the Marine Coating

The miscibility of HDES in biocide-free marine coating (CuO_2_ and Cu-free coating Hempel Portugal, S.A.) was evaluated by mixing the HDES with the coating and assessing its dissolution. A high amount of HDES was used to ensure total dissolution without phase formation. The coating with the incorporated HDES was applied on microscope slides for further analysis using an inverted optical microscope (Axio Vert A1, Zeiss, Oberkochen, Germany). Several mixtures with different concentrations of HDES Men/OL (1:1), namely 5, 15, 25, and 50 mg/mL, were incorporated in the CuO_2_ and Cu free coating for antimacrofouling tests, as explained in Section 3.2.

### 2.3 HDES Characterization

#### 2.3.1 Polarized Optical Microscopy

Optical characterization of the HDES systems was performed at room temperature using the transmission mode of a BX-51 polarized optical microscope (Olympus, Tokyo, Japan) connected to an Olympus KL2500 LCD cold light source. One drop of each HDES system was placed on the respective glass microscope slide for observation. Images were obtained using the equipped camera (Olympus SC50) and Olympus Stream Basic 1.9 software (Olympus, Tokyo, Japan).

#### 2.3.2 Nuclear Magnetic Resonance

NMR experiments were performed using a 400 MHz Bruker ADVANCE II instrument. Mestrenova 12.0 software (Mestrelab Research, Santiago, Spain) was used for spectral processing and analysis. The HDES and raw materials were dissolved (30 mg/mL) in dimethyl sulfoxide-*d*_6_ (DMSO-*d*_6_, 99.9 at. % D, LOT. STBH4385, Sigma-Aldrich). All the experiments were performed when the systems were in equilibrium, and no further changes in their properties were observed.

#### 2.3.3 Attenuated Total Reflection–Fourier Transform Infrared Spectroscopy

Spectroscopic analysis was performed by Fourier transform infrared spectroscopy using a Thermo Scientific spectrometer (Class 1 Laser Product Nicolet 6100, San Jose, CA) operating in attenuated reflection (ATR). Spectrum acquisition was performed using PerkinElmer Spectrum IR Version 10.6.2 software.

#### 2.3.4 Cytotoxicity Assessment

The cytotoxic effect was assessed using a confluent and differentiated human keratinocyte immortalized cell line (HaCat). The HaCaT cell line (German Cancer Research Center (DKFZ), Germany) was cultured according to the manufacturer’s instructions in Dulbecco’s modified Eagle medium (Sigma-Aldrich), supplemented with 10% heat-inactivated fetal bovine serum (FBS, Corning, USA) and 1% penicillin–streptomycin solution (PS, Corning, NY, USA). The cell culture was maintained in a humidified atmosphere at 37 °C with 5% CO_2_. The cytotoxicity assay was performed in accordance with ISO/EN 10993 guidelines. HaCat cells were seeded into 96-well plates at a density of 4.5 × 10^4^ cells/well and allowed to grow for 72 h. Cells were incubated with culture medium (control) and with different HDES concentrations diluted in the culture medium. After 24 h of exposure to HDES, the cells were washed twice with PBS, and cell viability was assessed using a CellTiter 96 AQueous One Solution Cell Proliferation Assay (Promega, Madison, Wi, USA) containing MTS (3-(4,5-dimethylthiazol-2-yl)-5-(3-carboxymethoxyphenyl)-2-(4-sulfophenyl)-2*H*-tetrazolium). Briefly, 100 μL of the viability reagent was added to each well at a 1:10 dilution and incubated for 3 h. The absorbance was measured at 490 nm using a microplate reader (VICTOR NivoTM, PerkinElmer, Waltham, MA, USA), and cell viability was expressed in terms of the percentage of living cells in relation to the control. Three independent experiments were performed in triplicate. The mean effective concentration (EC_50_) was obtained using best-fitting trend lines.

#### 2.3.5 Antimacrofouling Effect of the Men/OL (1:1) System Applied in Marine Coating

HDES were dissolved in biocide-free marine coating as described in 2.1 to study the antifouling effect of the eutectic system. These mixtures were used to paint glass plates (7 × 8 cm). As controls, biocide-free marine coating, copper-enriched marine coating (copper content: 25–50% copper(I) oxide (Cu_2_O) and 1–3% of metallic Cu, Hempel Portugal, S.A.), and biocide-free marine coating with ivermectin incorporation (13 mg/mL) were used. The coated plates with different concentrations of HDES were distributed in duplicate in the respective marine fouling organisms’ tanks with a volume of 10 L containing synthetic saline water. The marine organisms were randomly allocated on the plates. Adult (mature) specimens of similar size were the selection criteria for the organism’s collection. In this assay, mussels [*N* = 12; 3.84 ± 0.58 cm (length)] and *Patella vulgata* [*N* = 4; 54.33 ± 4.5 mm (length)] were used and added to each plate. These organisms were selected because they have strong adhesion power to surfaces, thus enhancing the understanding of the antifouling capacity of HDES. The limpets, like mussels, were collected manually from a region considered pristine at Guincho (Cascais, Lisbon, Portugal) and were acclimatized in the laboratory in a tank (200 L) with continuous aeration (>6 mg/L of dissolved oxygen) for at least 96 h. Before the beginning of the bioassays, they were cleaned to remove impurities.

The tanks were in a closed circuit and continuous aeration system, keeping the oxygen rate above 6 mg/L. The mussels and limpets were fed every 48 h with about 2 mg of *Chlorella* seaweed (Superfood Shine, Portugal) previously dissolved in synthetic saline water. The tanks were monitored daily for pH (7.78 ± 0.18), temperature (21 °C ± 1), and salinity (33 ± 1 g/L), and the experimental conditions were renewed every 48 h.

The effect of the HDES incorporated in the marine coating on the adhesion of organisms was monitored over time, and the behavior of the tested marine organisms in terms of their ability to adhere to the surface of the plates with respect to the respective control coatings and HDES concentrations was observed. The survival rate was determined by comparing the number of organisms alive (day after day) with the total number of organisms present at the beginning of the assay.

At the end of the experimental assay, marine organisms were sampled, weighed, and immediately frozen at −80 °C for further biochemical analysis.

##### 2.3.5.1 Quantification of Menthol/Oleic Acid (1:1) in Water

Water was collected after 48 h of immersing the coated glass plates and before replacing the water. The quantification of HDES in the water was performed through the quantification of the two components in the medium. In the case of menthol, the quantification was performed using GC-FID, and oleic acid was quantified using HPLC (DIONEX Summit (Sunyvale, USA). In detail, for the quantification of menthol, an extraction from water with *n*-hexane (99%, Carlo Erba, France) was performed in a ratio of 1:1 GC-FID chromatographic separation was performed using a capillary column HP 5 ms (a length of 30 m, an internal diameter of 0.25 mm, and a film thickness of 0.25 μm) with a mobile phase composition of 5% phenyl and 95% of dimethylpolysiloxane. The equipment used was an Agilent 6890 GS Gas Chromatograph Series (Agilent Technologies, USA) equipped with a flame ionization detector (FID). The front inlet was split/splitless with electronic pressure control (EPC). The GS parameters are summarized in Table 1.

**Table 1:** GC-FID Parameters for Menthol Quantification

carrier gas	helium			
column oven temperature (°C) programmed	rate (°C/min)	temperature (°C)	hold time (min)	total time
	initial	40	1	1
	10	325	0	29.5
detector temperature (°C)	250°C			
injector temperature (°C)	325°C			
flow rate	1 mL/min			
injection volume	1 μL			
gas flow rate	H_2_: 30 mL/min			
	He: 5 mL/min			
	air flow: 300 mL/min			

For the quantification of oleic acid, a Supelco Discovery HS-C18 4.6 × 250 mm column was used. In this case, the eluent was a mixture of acetonitrile/methanol/hexane in a ratio of 90:8:2 with 2% of ethanoic acid at a flow rate of 1 mL/min; the injection volume was 230 μL, and the detection was performed at 208 nm. The quantification conditions were operated at room temperature. This method was adapted from the article published by Guarrasi and her collaborators.^23^

The indirect calculations were performed based on the molar ratio between the compounds, i.e., the number of moles of menthol is equal to the amount of oleic acid, and the concentration was determined according to this ratio.

### 2.4 Biochemical Assays

#### 2.4.1 Sample Treatment

Immediately before starting the assay for testing the antimacrofouling capacity of HDES Men/OL (1:1), mussels (*N* = 5) and limpets (*N* = 3) were sampled, and several oxidative stress biomarkers were analyzed for T0. Salinity and pH (33 ± 1 g/L and 7.78 ± 0.18, respectively) were measured daily, and the experimental conditions were renewed every 48 h. The organisms were exposed to the different HDES concentrations for 21 days. At each sampling period (7 days), five mussels exposed to each HDES concentration were sacrificed and stored at −80 °C.

The gills and digestive glands of each organism were removed, weighed, and placed in microtubes (1.5 mL). The organs were homogenized using a tissue homogenizer (Tissue Master 125, Omni, Kennesaw, GA, USA) in 2 mL of phosphate-buffered saline solution (PBS)^24^ stored at −40 °C at pH 7.4 ± 0.2. The homogenized samples were centrifuged for 10 min at 15,000*g* at 4 °C (VWR, model CT 15RE from Hitachi Koki Co., Ltd., Tokyo, Japan). The supernatants were collected and stored at −80 °C until further analysis. The results of all the biochemical toxicity assays were normalized in relation to the total cytosolic protein concentration (nmol/min/mg) determined by the Bradford method.^25^

#### 2.4.2 Bradford Assay

The total protein concentration of the samples was determined using the Bradford method.^25^ Standards were prepared by serial dilution of a BSA (bovine serum albumin) stock solution in PBS to build a calibration curve ranging from 0 to 4 mg/mL of BSA. Subsequently, 20 μL of either the BSA standard or the samples and 180 μL of the Bradford reagent were added to a 96-well microplate (Greiner, Bio-One GmbH, Frickenhausen, Germany); both the standards and the samples were analyzed in duplicate. Absorbance was read at 595 nm using a microplate reader (Synergy HTX, BioTek, USA). The protein concentration of the samples (mg/mL) was used to normalize the results obtained in the following toxicological tests, i.e., the toxicity results are expressed in relation to the cytosolic protein content of the samples in nmol/min/mg cytosolic protein.

#### 2.4.3 Glutathione-*S*-transferase

Glutathione-*S*-transferase activity was determined according to the method described by Habig et al.,^26^ adapted for microplates. All chemical reagents were purchased from Sigma-Aldrich (USA). This method is based on the increased absorption at 340 nm that follows the formation of a conjugate between GSH and 1-chloro-2,4-dinitrobenzene (cDNB). To perform this assay, a substrate mixture was prepared by combining GSH 200 mM and cDNB 100 mM in phosphate buffer (PBS). Then, 180 μL of this solution was added to 20 μL of sample GST into each well of the 96-well microplate (Greiner Bio-one, Austria). The enzyme activity was determined by recording absorbance at 340 nm every min for 6 min using a microplate reader (Synergy HTX, BioTek, USA). The increase in absorbance per min was estimated, and the reaction rate was calculated using a cDNB extinction coefficient of 0.0053 μM^–1^. Equation 1 represents the formula for calculating GST activity. The GST activity was obtained after normalization with the cytosolic protein mass (nmol/min/mg cytosolic protein) determined in the Bradford assay (Section 2.4.2).
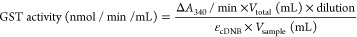
1

Equation 1. Calculation of glutathione-*S*-transferase (GST) activity.

#### 2.4.4 Superoxide Dismutase

The determination of superoxide dismutase followed the nitroblue tetrazolium (NBT) reduction method adapted from Sun et al.^27^ In this method, superoxide radicals (O_2_^•–^) are generated by the reaction of xanthine with xanthine oxidase (XOD), and NBT is reduced to formazan, which can be evaluated spectrophotometrically at 560 nm. SOD competes with NBT for the dismutation of O_2_^•–^, inhibiting its reduction. The level of inhibition is used as a measure of SOD activity. The assay was performed using 96-well microplates (Greiner Bio-one, Austria), adding to each well 200 μL of phosphate buffer 50 mM (pH 8.0), 10 μL of xanthine 3 mM (Sigma-Aldrich, Germany), 10 μL of 0.075 mM NBT (Sigma-Aldrich, Germany), 10 μL of 3 mM EDTA (Riedel-Haen, Germany), 10 μL of xanthine 3 mM (Sigma-Aldrich, Germany), 10 μL of NBT 0.75 mM (Sigma-Aldrich, Germany), and 10 μL of the sample. The reaction started with the addition of 10 μL of XOD (Sigma-Aldrich, Germany); the absorbance was recorded every two min for a total of 26 min at 536 nm using a plate reader (Synergy HTX, BioTek, Winooski, VT, US). Negative controls included all the mixture components except the sample, producing a maximum peak in absorbance at 536 nm, which allowed determining the percent inhibition per min caused by SOD activity (eq 2). Results are expressed in % of inhibition after normalization with the cytosolic protein mass.
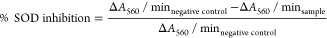
2

Equation 2. Calculation of % inhibition of superoxide dismutase (SOD).

#### 2.4.5 Catalase (CAT)

Catalase activity was determined according to the spectrophotometric method described by Beers and Sizer,^28^ adapted for 96-well microplates (UV-Star, Greiner-bio- one, Germany). In this assay, the rate of absorbance reduction was measured at 240 nm due to the consumption of H_2_O_2_ by catalase. Briefly, a hydrogen peroxide substrate solution (0.036% w/w) was prepared in potassium phosphate buffer 50 mM (KH_2_PO_4_; pH 7.0 at 25 °C) (Sigma-Aldrich). Subsequently, 7 μL of sample, followed by 193 μL of substrate solution, were added to each well of the microplate. Absorbance was read at 240 nm on a microplate reader (Synergy HTX, BioTek, USA) every 13 s for 3 min. CAT activity was determined as shown in eq 3, measuring the absorbance per min [(Δ*A*_240_)/min] and using the H_2_O_2_ molar extension coefficient of 0.04 μM^–1^. Catalase activity was expressed in nmol/min/mg after normalization using the cytosolic protein mass.
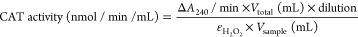
3

Equation 3. Calculation of catalase activity (CAT) 240 nm.

#### 2.4.6 Glutathione Peroxidase (GPx)

The determination of glutathione peroxidase activity followed the method of Lawrence and Burk,^29^ adapted for 96-well microplates. Briefly, 20 μL of each sample was added to each well of a 96-well microplate (Greiner Bio-one, Austria), followed by adding 120 μL of assay buffer (50 mM potassium phosphate buffer (pH 7.4, Sigma-Aldrich, Germany), EDTA 5 mM (Riedel-Haen, Germany), and 50 μL of the co-substrate mixture) to the microplate wells. The co-substrate mixture was composed of sodium azide 4 mM (Sigma-Aldrich, Germany), nicotinamide adenine dinucleotide phosphate 1 mM (NADPH, Sigma-Aldrich, Germany), glutathione reductase 4 U/mL (GSSG-reductase, Sigma, Germany), and reduced glutathione 4 mM (GSH, Sigma-Aldrich, Germany). The reaction was initiated by the addition of 20 μL of cumene hydroperoxide at 15 mM (Sigma-Aldrich, Germany), and the absorbance was read at 340 nm every min for a total of 6 min using a microplate reader (Synergy HTX, BioTek, Winooski, VT, US). The decrease in absorbance per min (Δ*A*_340_) was determined, and the reaction rate was calculated using the β-NADPH extinction coefficient (3.73 mM^–1^). Equation 4 represents the formula for calculating GPx activity, expressed in nmol/min/mg and normalized using the cytosolic protein mass.
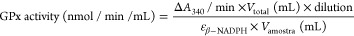
4

Equation 4. Calculation of glutathione peroxidase (GPx) activity.

#### 2.4.7 Lipid Peroxidation

The lipid peroxidation assay was determined using the thiobarbituric acid reactive species (TBARS) method.^30^ The TBARS method is based on the reaction of malondialdehyde (MDA), a lipid peroxidation product with thiobarbituric acid (TBA), which produces a compound that absorbs at 530 nm. To perform this assay, microtubes (1.5 mL) were used to prepare both the standards and the samples. Briefly, 5 μL of each sample were added to microtubes, followed by 45 μL of 50 mM monobasic sodium phosphate buffer. Then, 12.5 μL of 8.1% SDS (Sigma-Aldrich, Germany), 93.5 μL of trichloroacetic acid (Panreac, Spain) (20%), and 93.5 μL of thiobarbituric acid (Sigma-Aldrich, Germany) (1%) were added to each microtube. Tubes were vortexed, and subsequently, lids were pierced with a needle. Next, the samples and standards were incubated in boiling water (10 min at 100 °C). Immediately after this procedure, these were placed on ice for a few min to cool. Subsequently, 62.5 μL of MiliQ grade ultrapure water was added into each microtube. The microtubes were vortexed again. Triplicates of 150 μL of each microtube were added into each well of a 96-well microplate (Greiner Bio-one, Austria); absorbance was read at 530 nm using a microplate reader (Synergy HTX, BioTek, Winooski, VT, US). To quantify lipid peroxides, a calibration curve was constructed with MDA solution in MiliQ ultrapure water within a range from 0 to 0.1 μM. The results were expressed in relation to the total amount of cytosolic protein in the samples (pmol/mg cytosolic protein).

### 2.5 Scanning Electron Microscopy

The samples of the coatings with different concentrations of HDES that coated the submerged plates were gently removed, and the adhered microorganisms to their surface were treated with a phosphate-saline buffer with 10% (v/v) formalin for 1 h and washed with deionized water. The adhered biofilm was then dehydrated by immersion in solutions with increasing concentrations of ethanol (20, 50, 70, 90, and 100%). Finally, samples were left to dry in a safety cabinet overnight and then gold-sputtered for SEM image acquisition (Carl Zeiss AURIGA CrossBeam (FIB-SEM)).

### 2.6 Data and Statistical Analysis

Statistical analyses were performed using Graph PadPrism 8 (Graph Pad Software Inc., San Diego, CA, USA) version 8.0. The data were expressed as mean ± standard deviation (SD). Statistical comparisons were analyzed by one-way ANOVA (in the case of parametric tests) or by the Kruskal–Wallis test (in the case of non-parametric tests), followed by Dunnett’s test of multiple comparisons. A significance level of *p* < 0.05 was considered.

## 3 Results and Discussion

### 3.1 Design of HDES

The design of HDES is still a trial-and-error process due to the lack of knowledge on the interactions established between the system counterparts, and therefore, it is necessary to carry out its preparation by experimentally mixing different molar ratios. Table 2 summarizes the eutectic systems prepared and the resulting visual aspects of the different formulations at room temperature (RT), indicating that completely clear liquids were successfully obtained at various tested molar ratios. The liquid phase is a strong indication of the existence of intermolecular interactions between the compounds that form the HDES.^31,32^

**Table 2:** Studied HDES: Identification of Prepared HDES Systems, Respective Molar Ratios, and States at Room Temperature

name	HDES abbreviation	HDES molar ratio	visual aspect at RT
menthol/oleic acid	Men/OL	1:1	translucent liquid
menthol/3-hydroxybutyric acid	Men/HB	2:1	translucent liquid
menthol/3-hydroxybutyric acid	Men/HB	3:1	translucent liquid

### 3.2 Assessment of the Miscibility of HDES in the Marine Coating

**Figure 1:**
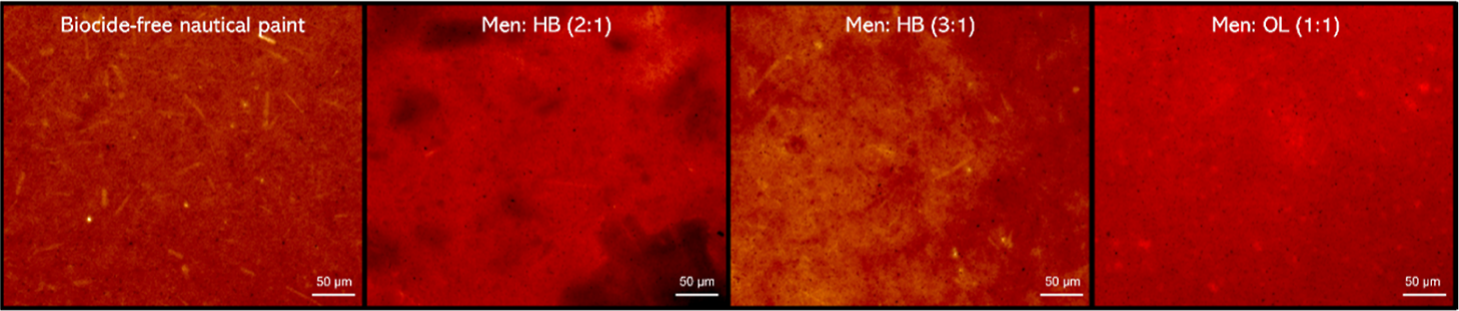
Study of the behavior of the prepared HDES dissolved in marine coating. Optical microscopy images obtained with ×200 magnification.

To understand the miscibility of the prepared HDES dissolved in the marine coating, several HDES incorporated in the biocide-free marine coating were analyzed by optical microscopy (Figure 1). Compatibility between the HDES and the coating was verified, and no resemblance to an emulsion (phase separation) was observed under the optical microscope. Therefore, the solutions appeared to be homogeneous and well distributed.

### 3.3 Physicochemical Characterization of HDES

#### 3.3.1 HDES POM Analysis

**Figure 2:**
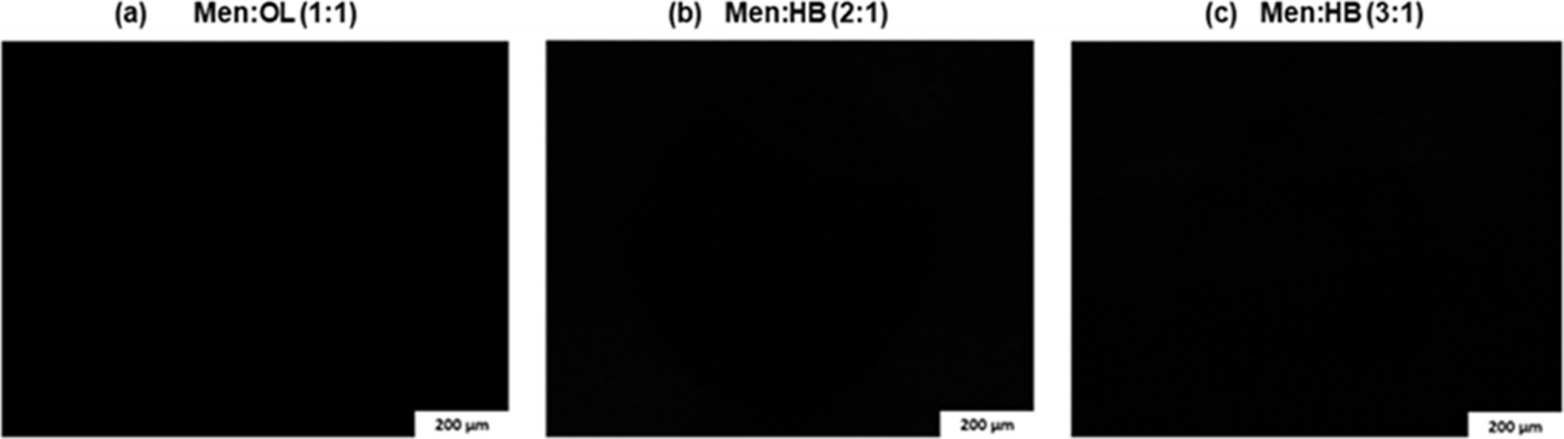
HDES POM. POM micrographs of HDES systems studied at room temperature. (a) Men/OL (1:1); (b) Men/HB (2:1); and (c) Men/HB (3:1).

Polarized optical microscopy was used to detect the existence of different phases in the prepared HDES, by assessing the existence of crystal-like structures in the eutectic mixture.^32^ When a uniform liquid mixture is present, the image is black (Figure 2).

Thus, the POM micrographs corroborated the naked eye visual and optical microscopy observations, as no crystal-like formation was distinguished, which is indicative of homogeneous HDES systems at RT.

#### 3.3.2 HDES NMR Analysis

Owing to the gap in understanding the interactions between the compounds, the supramolecular arrangement of HDES was further evaluated using ^1^H NMR, allowing us to study the intermolecular interactions of the atoms involved and confirm the ratios of the system’s counterparts. ^1^H NMR spectra for the isolated components and eutectic systems are presented in Figures S1 and S2, respectively. In what concerns Men/HB eutectic systems, in both molar ratios, the spectrum of the individual component menthol (Figures S1a) presents a well-defined doublet with respect to its hydroxyl group (δ = 4.27–4.28 ppm), whereas in the HDES spectrum, a broader singlet was observed (Figure S2a,b). The establishment of hydrogen bonds is evidenced by the widening of the chemical shift, corresponding to the proton of the hydroxyl group of the menthol in the system, compared to what occurs in the spectrum of the initial menthol (Figure S1a).

Another evidence of the establishment of hydrogen bonds between these molecules is the signal of the proton attached to the carbon that bonds to the OH group of menthol (H-a) (Figure S1a). In the ^1^H NMR spectrum of the individual component menthol compound (Figure S1a), this signal presents ^1^H resonance at a chemical shift of 3.13–3.18 ppm, being, as expected, a multiplet. However, in the ^1^H NMR spectra of the studied eutectic systems [Men/OL (1:1), Men/HB (2:1), and Men/HB (3:1)] (Figure S2a–c, respectively), besides no detectable shift, the signals are no longer a well-defined multiplet, which further suggests that the H-a of menthol was affected by hydrogen bond interactions between the parent molecules. This difference was even more noticeable in the Men/OL (1:1) spectrum (Figure S2c). These results suggest the establishment of interactions between the compounds of each system via hydrogen bond formation, corroborating the results obtained by the POM analysis. ^1^H–^1^H-nuclear Overhauser enhancement (NOESY) spectroscopy was also performed, and the results are included as Supporting Information (Figure S3).

The spectra of the Men/OL (1:1) system showed interaction between the −OH group of oleic acid and the H-a proton of menthol (bonded to the carbon to which the −OH of menthol is attached), as seen in the previously analyzed ^1^H NMR spectra (Figure S3a). Moreover, the interaction between the −OH groups of the two compounds was verified (Figure S3a).

In what concerns the spectra for the Men/HB (2:1) and Men/HB (3:1) systems, interactions between the −OH groups of the two compounds were verified (Figure S3b), along with interactions between the −OH groups of menthol with 3-hydroxybutyric acid protons (Figure S3b,c). The observed results may be compatible with the formation of a supramolecular network, which is characteristic of HDES, as seen in the ^1^H NMR spectra.

The Men/OL (1:1) system was considered for the antifouling and toxicity assays, due to the fact that the cost of oleic acid is much lower when compared to that of 3-hydroxybutyric acid, which will have an impact on a potential industrial application.

### 3.4 Cytotoxic Potential of the Formulated HDES

The synergistic/additive effects can, in some cases, lead to more toxic systems compared to their constituents. In this scenario, the cytotoxic effect of the Men/OL (1:1) system on a human keratinocyte cell line (HaCat), acting as a preliminary safety indicator, was evaluated by calculating the median effective concentration (EC_50_). Since the present study aims at the application of HDES in marine coatings, it will exert contact, both in humans and marine organisms, through the dermal route as well as the oral route. Therefore, the evaluation of the cytotoxic effect in epidermal cells (HaCat cell line) was performed, and the results are presented in Table 3.

**Table 3:** Cytotoxicity Assay and EC_50_ Values for the Men/OL (1:1) System Using a Human Keratinocyte Cell Line (HaCat)^a^

HDES	EC_50_ values (mg/mL)
Men/OL (1:1)	0.83 ± 0.39

a The results were obtained from three independent experiments performed in triplicate as the mean ± standard deviation (SD).

No cytotoxic studies have been described in the literature for this eutectic system. However, in a reported study, which used systems also composed of menthol and natural organic acids, namely, Men/lauric acid (4:1), Men/myristic acid (8:1), and Men/stearic acid (8:1), the authors hypothesized that the system’s cytotoxicity decreased with the presence of a saturated fatty acid in its composition. They reported an EC_50_ value of 0.92 ± 0.05 mg/mL for Men/lauric acid (4:1), which is close to that of Men alone, and a lower cytotoxicity for Men/myristic acid (8:1) and Men/stearic acid (8:1).^19^ It is interesting to highlight that the cytotoxicity of these eutectic systems appears to be related to the carbon chain size of the fatty acid, where a higher 18-carbon chain, such as in OL, seems to decrease cytotoxicity toward the tested cell line.

### 3.5 Antimacrofouling Potential of the Men/OL (1:1) System

The effect of the HDES incorporated in the marine coating on the adhesion of organisms was monitored over time, and the behavior of the tested marine organisms in terms of their ability to adhere to the surface of the plates with their respective coatings and HDES concentrations was observed.

**Figure 3:**
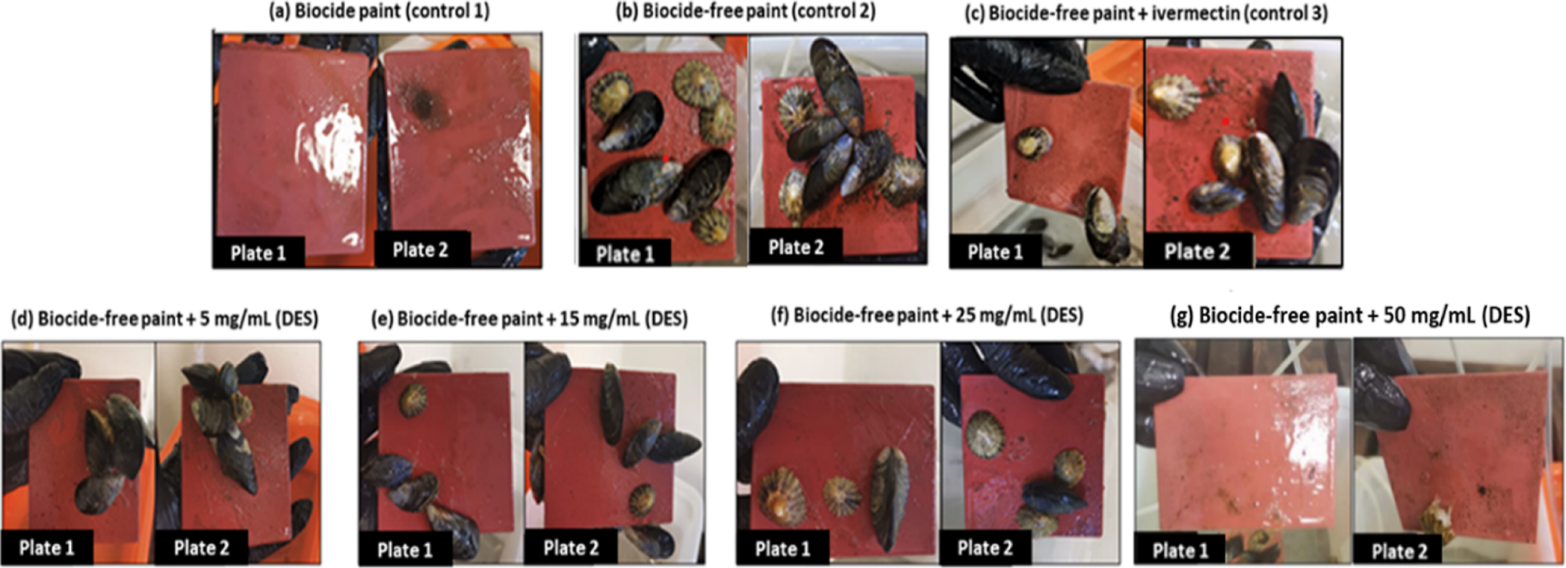
Antimacrofouling effect of the Men/OL (1:1) system applied to the marine coating. Study of several concentrations of HDES and analysis of the evolution of the fouling process exerted by marine organisms (*Mytilus edulis* and *Patella vulgata*)—day 7. The image shows two test plates present in the respective tanks (not technical replicates).

**Figure 4:**
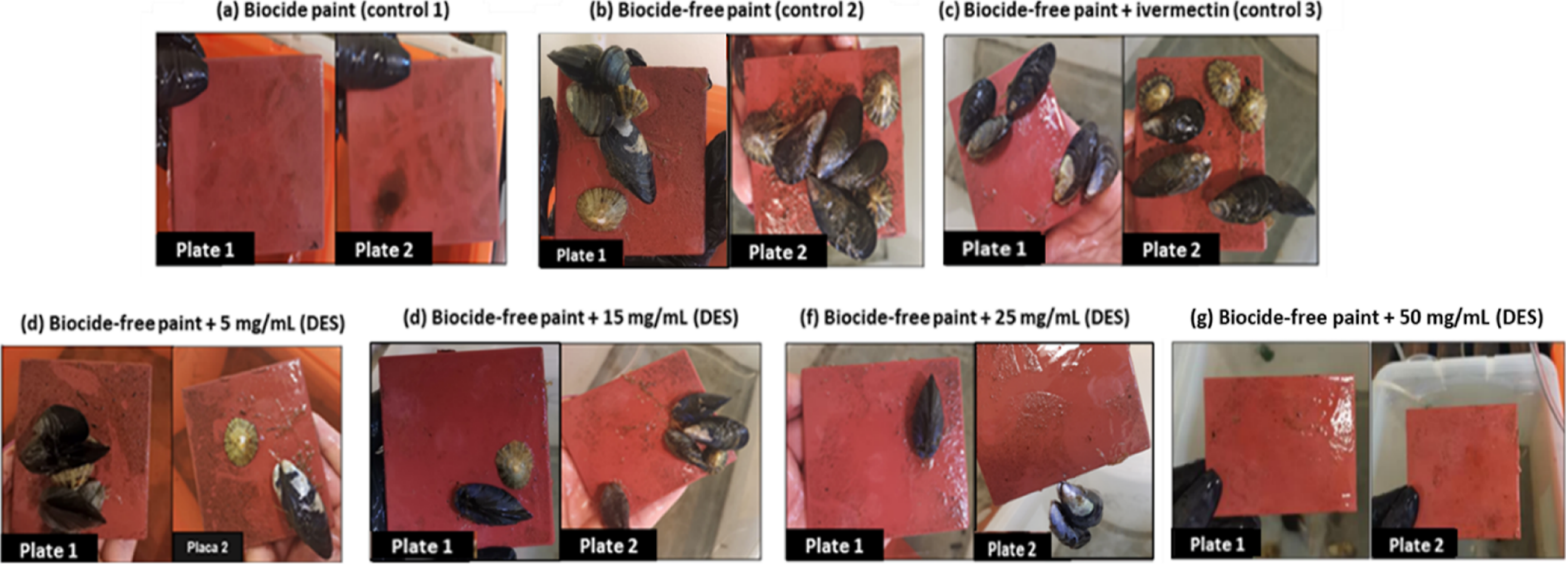
Antimacrofouling effect of the Men/OL (1:1) system applied in the marine coating. Study of several concentrations of HDES and analysis of the evolution of the fouling process exerted by marine organisms (*Mytilus edulis* and *Patella vulgata*)—day 10. The image shows two test plates present in the respective tanks (technical replicates).

Copper-enriched coating was used as a control in order to compare the effects on marine biota between this commercial coating and the coatings with dissolved HDES Men/OL (1:1). Several concentrations of HDES were tested in biocide-free coating, namely 5, 15, 25, and 50 mg/mL, to understand if the eutectic system demonstrates the ability to potentiate antifouling activity in the biocide-free coating without the need to use copper oxide and metallic copper. Biocide-free coating with the antifouling agent ivermectin was also used as a control for comparison purposes. The assays were performed for 10 days, when the total mortality of marine organisms was verified in the control with the copper-enriched coating. Figures 3 and 4 display the results obtained for the Men/OL (1:1) system antimacrofouling assay at days 7 and 10, respectively.

The plates covered with copper-enriched coating (Figures 3 and 4a) always demonstrated the absence of adhered microorganisms. However, this coating was revealed to be toxic to the organisms, causing their death.

The plates with biocide-free coating (Figures 3 and 4b) and biocide-free coating with the incorporation of ivermectin (Figures 3 and 4c) exposed the highest level of macrofouling. Plates coated with the Men/OL (1:1) system at different concentrations incorporated in biocide-free coating revealed a lower degree of adhesion by the tested organisms.

During the assay, an increasing antimacrofouling effect of HDES Men/OL (1:1) was observed, and this effect is more noticeable as its concentration increases. It was also verified that limpets, known for their strong adhesion ability, exhibited an increasingly weak adhesion with increasing HDES concentration and with the course of the assay, as many of these organisms ended up slipping off the plates during the handling procedure. It should be noted that on the last day of the assay (day 10), at the highest concentration (Figure 4g), no microorganisms were adhered to the plates. The Men/OL (1:1) system revealed an efficient antifouling activity at the concentration of 50 mg/mL (Figures 3 and 4g). Despite ivermectin being described as having antimacrofouling properties, the studied HDES [Men/OL (1:1)] demonstrated to be more effective as an antimacrofouling agent.

**Figure 5:**
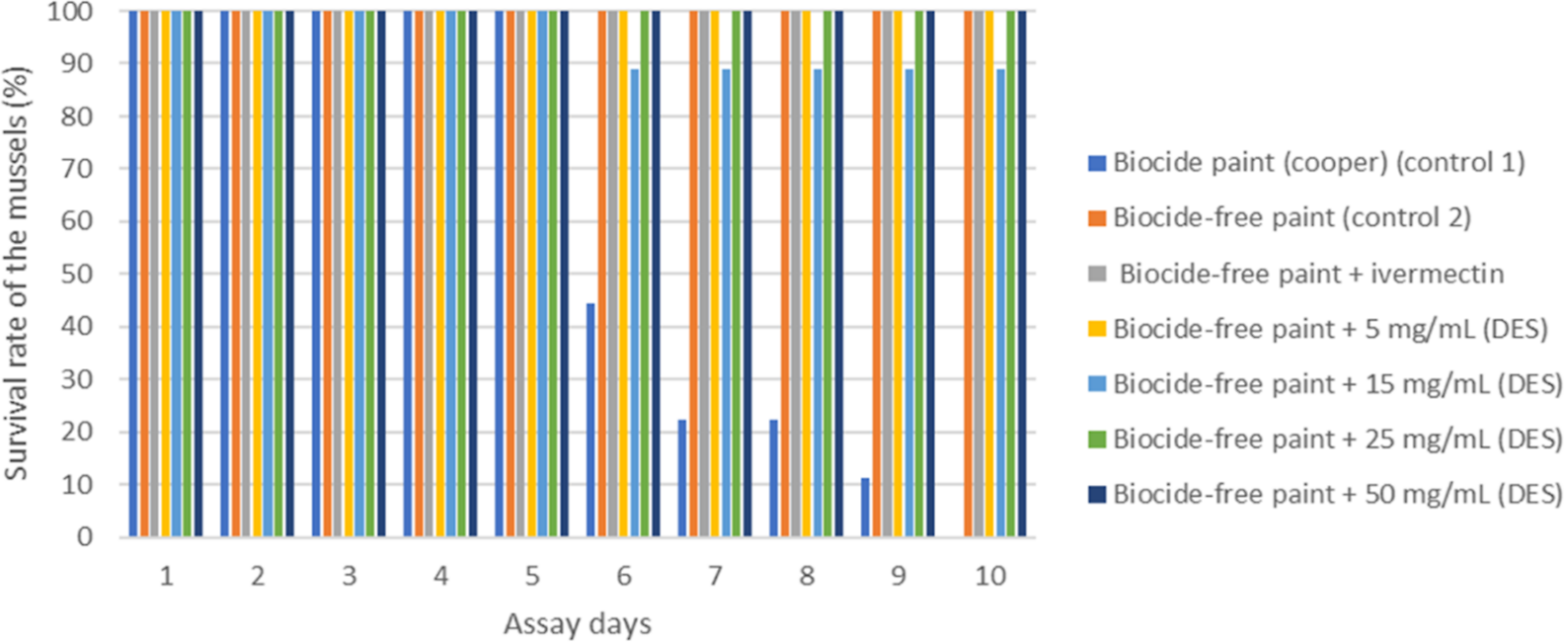
Analysis of the survival rate of mussels (*Mytilus edulis*) during the antifouling trial with the incorporation of HDES in marine coating. Nine mussels were present in each tank.

The survival rate of the marine organisms throughout the antifouling assay was also studied, and Figure 5 is representative for the case of the mussel *Mytilus edulis*.

The copper-enriched marine coating, despite having a strong antifouling effect, caused the death of all marine organisms during the test, being harmful to these marine species. The hydrophobic eutectic system Men/OL (1:1) showed an antifouling effect at different tested concentrations, with the highest concentrations (25 and 50 mg/mL) showing the best ability to minimize marine organisms’ adhesion. The presence of the eutectic system in the biocide-free coating demonstrated to potentiate its antifouling action while not negatively affecting marine species as much as the CuO_2_-enriched coating. Several studies showed that ivermectin was a promising substitute for marine antifouling. In the present study, the eutectic system demonstrated higher antifouling capacity compared to ivermectin even at its lowest concentration (5 mg/mL). The concentration of ivermectin in the coating corresponded to 13 mg/mL, which is within the concentration range of the eutectic system. It should be noted that ivermectin, like our eutectic system, is practically insoluble in water. The results emphasize the potential of the prepared HDES for antifouling coating applications.

The fact that the Men/OL (1:1) eutectic system presents antifouling properties, combined with its harmless effect on the marine ecosystem, makes it a potentially sustainable candidate as an antifouling agent with more beneficial effects on the environment, thus potentially mitigating marine biofouling, allowing the survival of marine species, and not introducing environmental imbalances.

#### 3.5.1 Quantification of Menthol/Oleic Acid (1:1) in Water

The quantity of menthol and oleic acid which leached from the coated paint to the water after 48 h is presented in Table 4. The concentrations of menthol and oleic acid were expressed in μg/L. This concentration represents the quantity of menthol and oleic acid which leached from the coated paint to the water.

**Table 4:** Concentration of Menthol and Oleic Acid Present in the Water after 48 h

	menthol (μg/L)	oleic acid (mg/L)	oleic acid (μg/L)^b^
biocide-free paint + 2 mg/mL DES	159.58	Nd^a^	288.44
biocide-free paint + 5 mg/mL DES	22.12	Nd^a^	39.99
biocide-free paint + 15 mg/mL DES	9.43	Nd^a^	17.04
biocide-free paint + 25 mg/mL DES	0.00	Nd^a^	0.00
biocide-free paint + 50 mg/mL DES	22.73	Nd^a^	41.08

a nd—not detected.

b Indirect calculation.

After 48 h, it was detected that there was a low concentration of menthol in the water, and no oleic acid was detected. This may be due to the different instruments’ detection limits; the detection limit of the method based on HPLC used to quantify oleic acid is much lower than the method based on GC-FID used to quantify menthol. For this reason, the quantity of oleic acid was also calculated indirectly, based on menthol quantification, knowing the molar ration between the two components, and assuming that these leaches are in the same proportion. The concentration of menthol and oleic acid in the water is extremely low when compared with the concentration in the glass, and for this reason, we can conclude that the majority of HDES did not leach. Thus, we may assume that the quantity of HDES that was maintained in the paint is approximately the same as at the beginning of the experiment.

### 3.6 Biochemical Assays in Marine Organisms

During the exposure period, water quality parameters remained stable, namely pH and temperature (7.78 ± 0.18 and 22.4 ± 0.7 °C, respectively). During the animal dissection, no loss of integrity and/or darkening of the gills and/or glands or limpets was observed.

**Figure 6:**
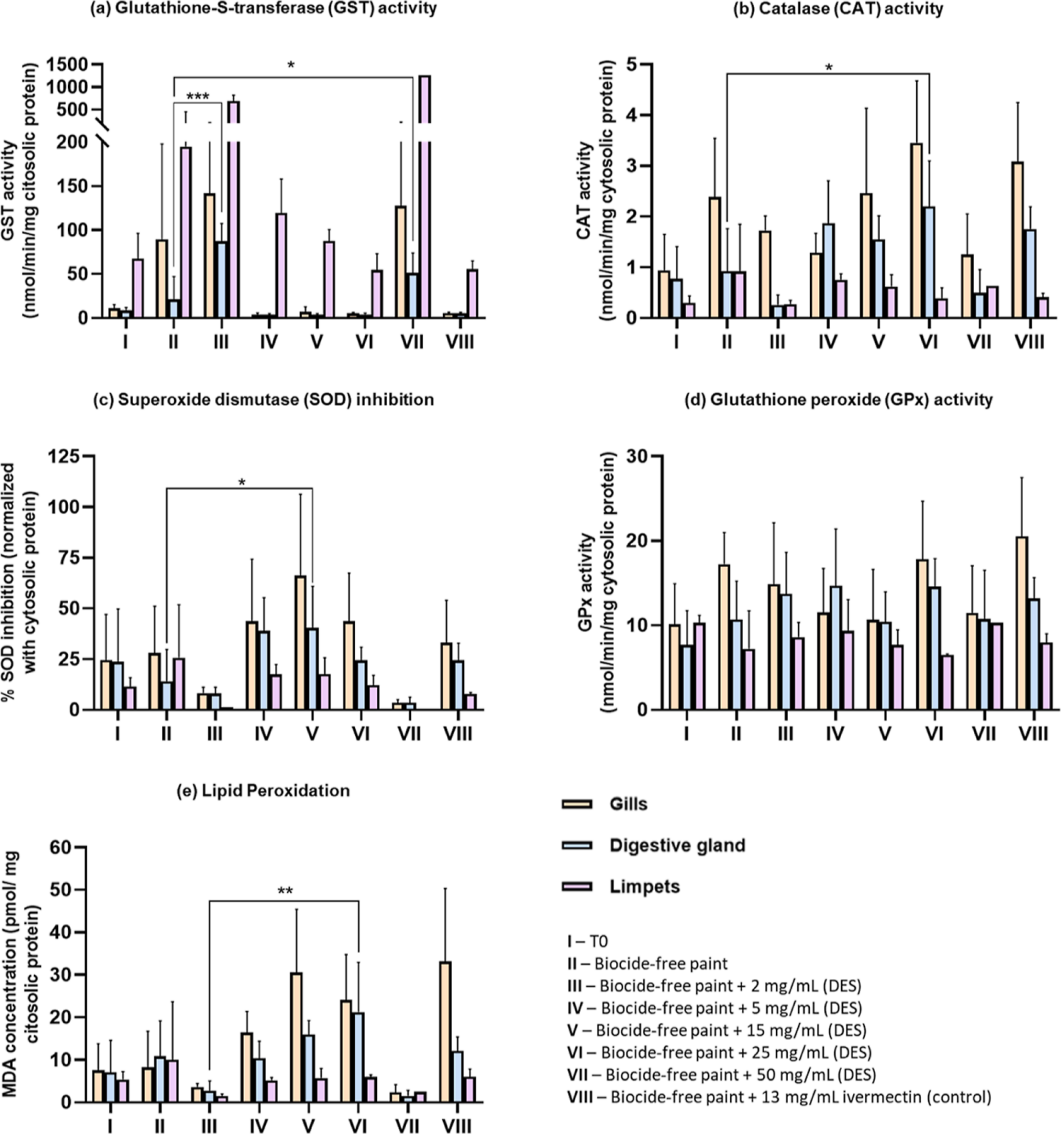
Biochemical assays in mussels (*Mytilus edulis*), gills, and digestive glands and limpets (*Patella vulgata*) from samples with the incorporation of different concentrations of the Men/OL system (1:1) in marine coating: (a) Glutathione-*S*-transferase (GST) activity, (b) catalase (CAT) activity, (c) superoxide dismutase (SOD) inhibition, (d) glutathione peroxidase (GPx) activity, and (e) lipid peroxidation. The data were expressed as mean ± standard deviation (SD). *—significant differences with *p* < 0.05, **—significant differences with *p* < 0.01.

The oxidative stress biomarkers (CAT, SOD, GPx, GST, and lipid peroxidation) used to assess the toxicity of the Men/OL (1:1) system are presented in Figure 6. All the significant variations verified in the respective biochemical assays are presented in Tables S1–S4.

The analyzed stress biomarkers suggest no significant (*p* > 0.05) variations between organisms (mussels and limpets) on arrival (T0) and control assays. The observed increase in some enzyme activities (e.g., SOD, CAT, and GST) and in LPO (MDA concentration) may suggest that the Men/OL (1:1) system can cause some oxidative stress in marine organisms. However, metabolic and enzymatic pathways of these organisms continue to properly function as cells respond by increasing enzyme levels, trying to fight oxidative stress by removing reactive oxygen species. However, the increase in MDA levels suggested damage to the cells, especially in mussels. Despite some variations in enzymatic activities verified in the respective graphics (Figure 6) at the highest concentration of HDES (50 mg/mL), the levels of SOD activity inhibition, catalase, and GPx activities remained low, meaning that there were no significant increases in the activity of these antioxidant enzymes in response to oxidative stress. In what concerns GST, the activity of this enzyme at the highest concentration of HDES increased, meaning that it is acting to detoxify the organism from toxic compounds and thus protecting the organism’s cells from toxicity. In turn, this increase can be a cause for the MDA levels decreasing significantly when comparing the control and the paint with higher HDES concentrations.

It should be noted that the observed significant individual variations may also be due to external factors, which may influence the results. Examples are the age/sex of the marine organism and concentration of the enzyme in a particular organ/organism, among others.

These results can be confirmed by lipid peroxidation, which measures biological damage caused by free radicals formed during oxidative stress. This assay is one of the most representative parameters of biological membrane damage by measuring MDA content as an indicator of lipid peroxidation. At the highest concentration of HDES, the levels of MDA are very low, which indicates that the Men/OL (1:1) system did not cause oxidative stress capable of affecting the enzymatic pathways of the tested organisms.

### 3.7 Antimicrofouling Potential of the Men/OL (1:1) System

**Figure 7:**
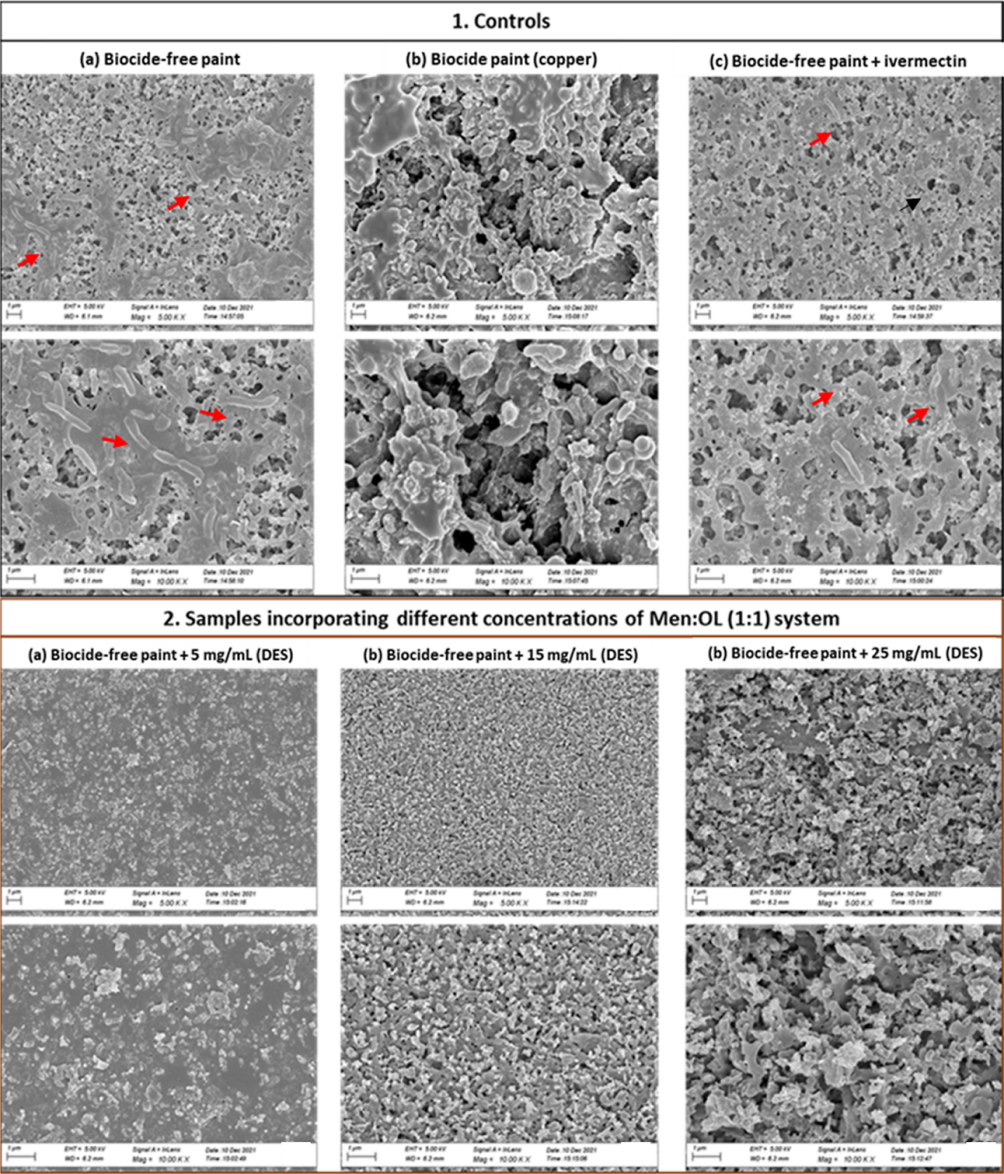
SEM images of the biofilm developed onto plates coated with the Men/OL (1:1) system studied intermediate concentrations and assessment of the antimicrofouling potential of this system: (1) SEM microscopy results for controls—(a) biocide-free coating; (b) copper-enriched coating; (c) biocide-free coating + ivermectin; (2) SEM microscopy results for samples incorporating different concentrations of the Men/OL (1:1) system: (a) biocide-free coating + 5 mg/mL (HDES); (b) biocide-free coating + 15 mg/mL (HDES), and (c) biocide-free coating + 25 mg/mL (HDES). The scale bar is 1 μm.

In the previous tests on the incorporation of the Men/OL (1:1) system in the marine coating, it was possible to determine its effectiveness in relation to macrofouling. Consecutively, the antimicrofouling effect of this HDES was also studied, as to prevent the formation of biofouling, it is extremely important to inhibit the formation of bacterial biofilms (antimicrofouling). In this sense, with SEM, we analyzed whether our HDES could inhibit/prevent the adhesion of bacterial cells without the use of antibiofilm agents and additional physical forces, thus demonstrating microfouling activity. SEM microscopy results for controls and samples of intermediate concentrations of HDES are presented in Figure 7.

Plates with a biocide-free coating revealed a significant number of bacteria. The copper-enriched coating also exhibited the presence of bacteria. Moreover, in the biocide-free coating with ivermectin incorporation (13 mg/mL), some bacteria were also observed. The most promising results were obtained for the coatings with various concentrations of HDES, where the absence of microbial cells was verified. With this result, the eutectic system also demonstrates to act as an antimicrofouling agent.

## 4 Conclusions

In the present work, we designed new eutectic systems based on natural molecules with antifouling activity which are compatible with marine coatings. The Men/OL (1:1) system showed excellent results as an additive of marine coatings, revealing a strong antifouling capacity at different tested concentrations while not inducing the death of the selected marine species. Moreover, it also showed improved results when compared to ivermectin, which is a biocidal agent used in coatings for macrofouling inhibition. The Men/OL system also demonstrated the ability to inhibit/prevent the adhesion of bacterial cells without the use of antibiofilm agents or additional physical forces, also acting at the microfouling level. Overall, HDES proved to be a promising antifouling candidate with strong experimental evidence. As a future perspective, in situ marine antifouling tests will be carried out. In this way, it would be possible to test the capacity of this HDES in a real marine environment for proof of concept and, thus, consolidate this formulation as an alternative marine fouling mitigation solution to address the fouling challenge.

## Supporting Information Available

^1^H NMR spectra of each DES and individual compounds; two-dimensional NOESY spectra of the HDES system; and all significant differences in respect to biochemical assays (PDF)
