# Supplementary material for: Hydrophobic DES Based on Menthol and Natural Organic Acids for Use in Antifouling Marine Coatings
Source: ACS Sustain Chem Eng. 2023 Jun 15;11(27):9989–10000. doi: 10.1021/acssuschemeng.3c01120 (PMC10337252; doi:10.1021/acssuschemeng.3c01120)
Supplement: Supplementary file 1 — sc3c01120_si_001.pdf [file sc3c01120_si_001.pdf]

Supplementary Information

Hydrophobic DES based on menthol and natural organic acids for use in antifouling marine coatings

Valente S.[a,b,c] , Oliveira F.[a] , Ferreira I.[a], Paiva A.[a], Sobral R. G.[b,c] , Diniz, M. [b,c], Gaudêncio S. P.*[b,c], Duarte A. R. C. *[a]

[a.] *LAQV-REQUIMTE, Chemistry Department, Faculty for Sciences and Technology, NOVA University of Lisbon, 2829-516 Caparica, Portugal.*

[b.] *Associate Laboratory i4HB – Institute for Health and Bioeconomy, NOVA School of Science and Technology, NOVA University Lisbon, 2819-516 Caparica, Portugal*

[c.] *UCIBIO, Chemistry and Life Sciences Departments, Faculty for Sciences and Technology, NOVA University of Lisbon, 2829-516 Caparica, Portugal.*

* Correspondence: - **Duarte A. R.** email: ard08968@fct.unl.pt
- **Gaudêncio S. P.** email: s.gaudencio@fct.unl.pt

Number of pages: 10
Number of tables: 3
Number of figures: 3

(a) Men

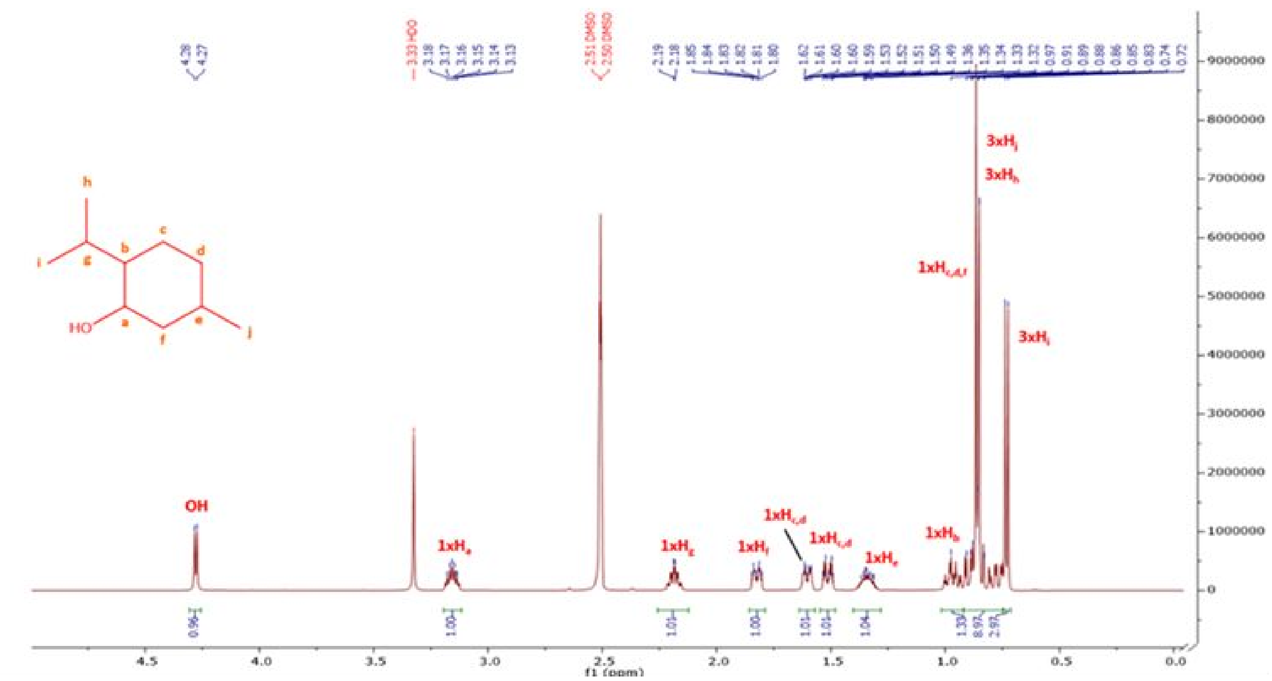
OH
1xHa
1xHg
1xHf
1xHc,d
1xHc,d
1xHe
1xHb
1xHc,d,f
3xHj
3xHh
3xHi
HO
f1 (ppm)

(b) HB

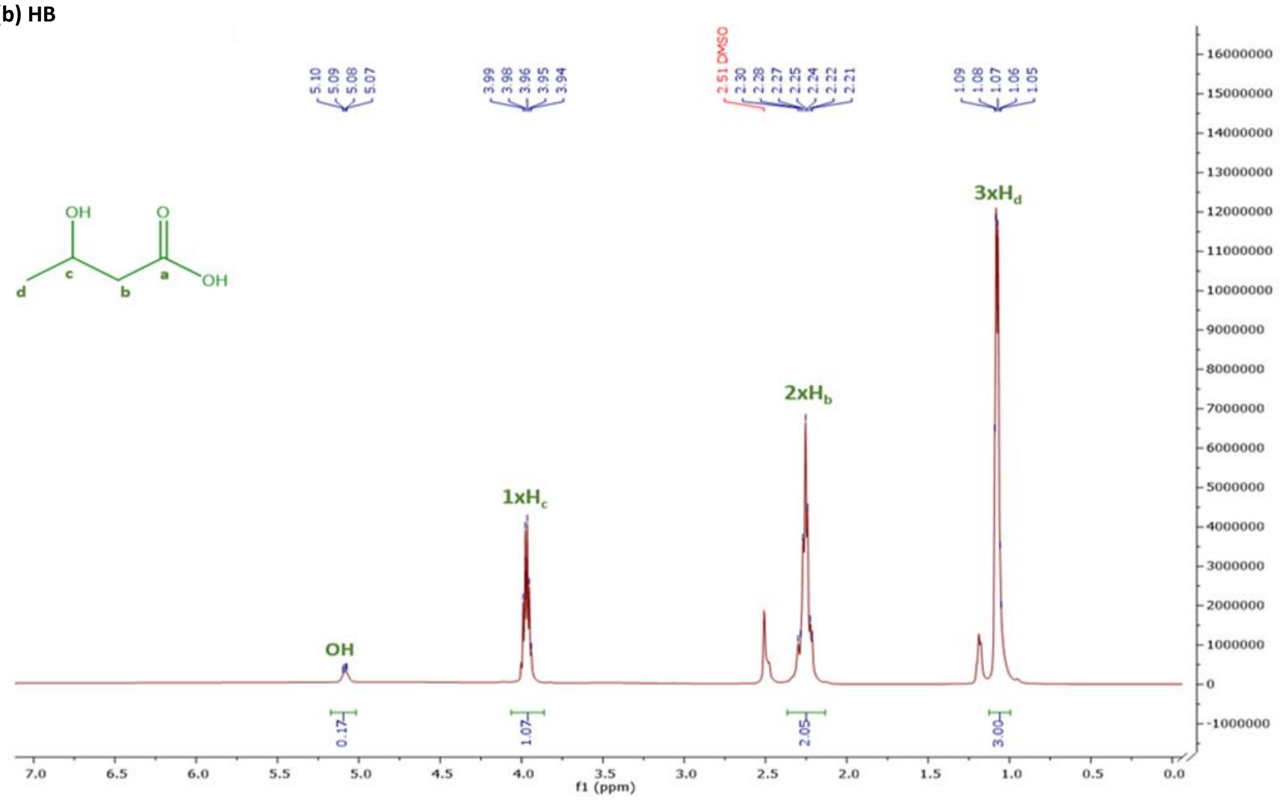
OH
1xHc
2xHb
3xHd
OH
O
OH
f1 (ppm)

(c) OL

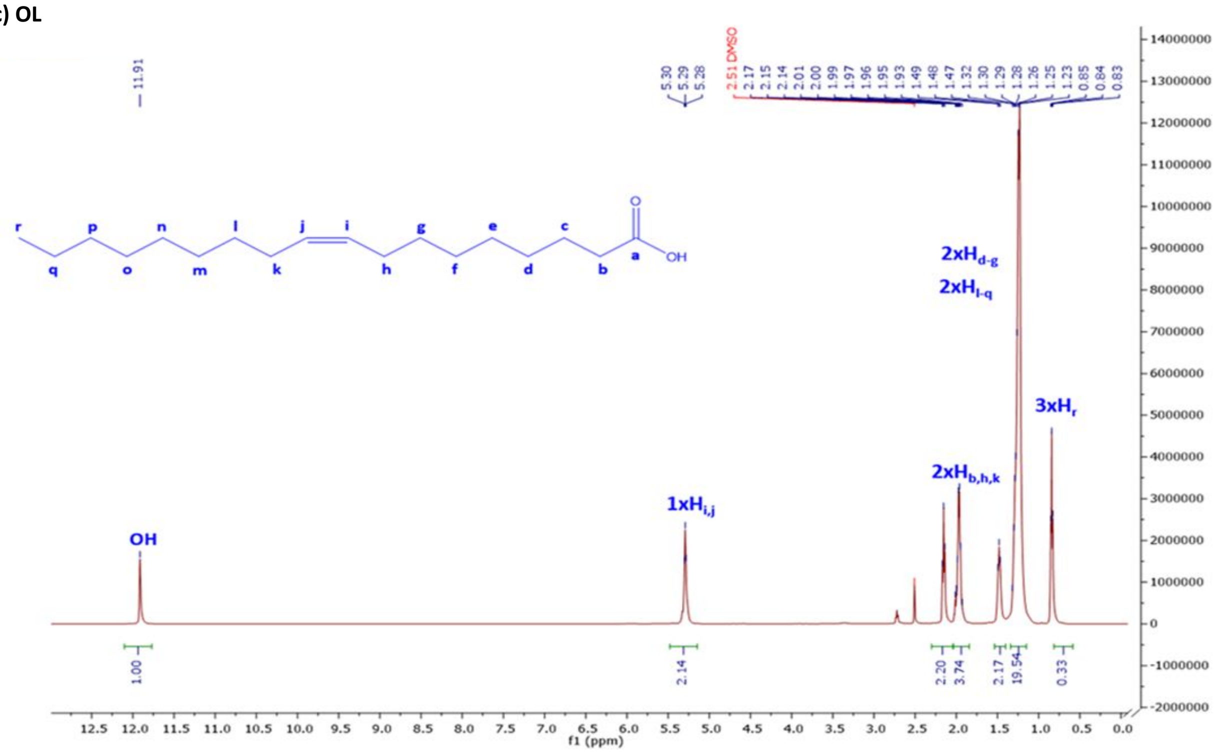

Figure S1. ^{1}H NMR spectra of (a) Men, (b) OL e (c) HB in DMSO-d6.

(a) Men:HB (2:1)

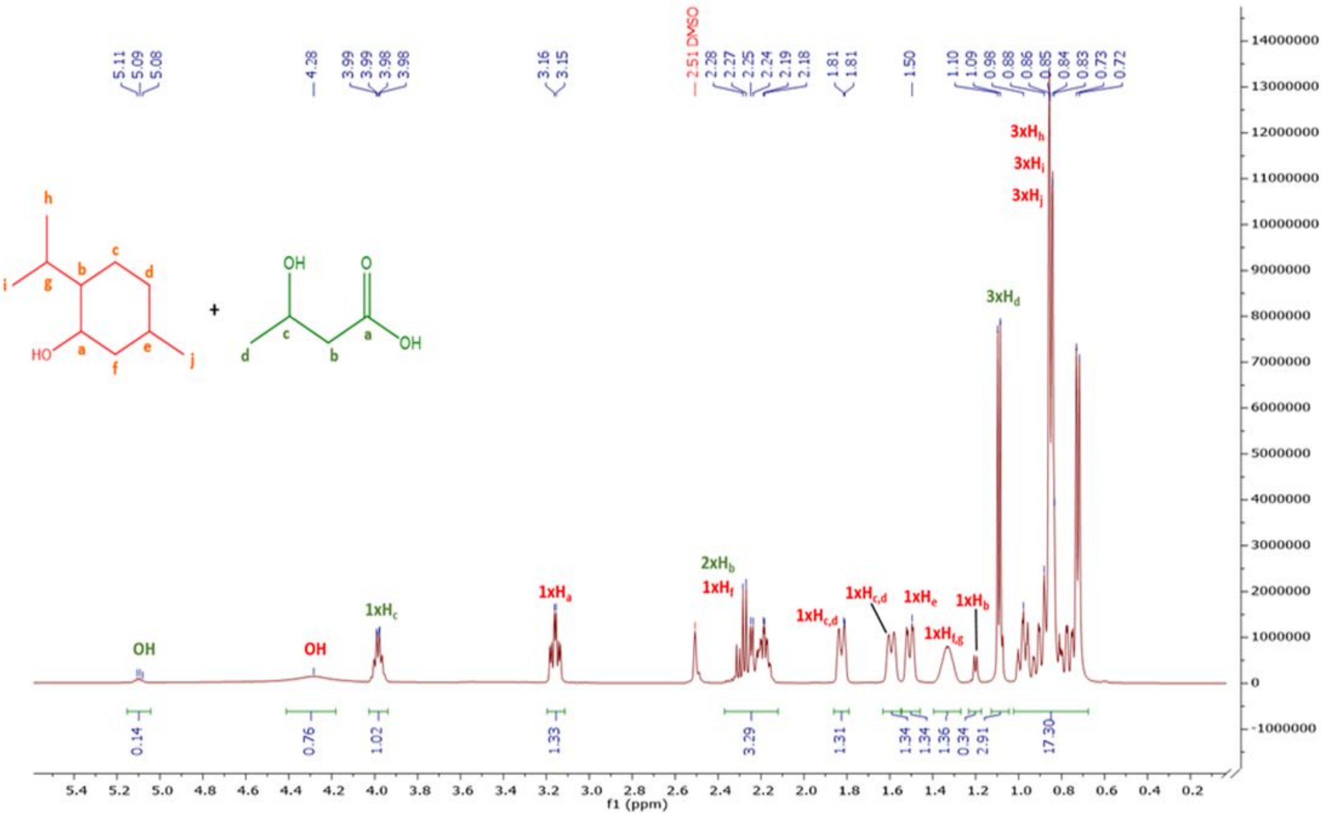

69

(b) Men:HB (3:1)

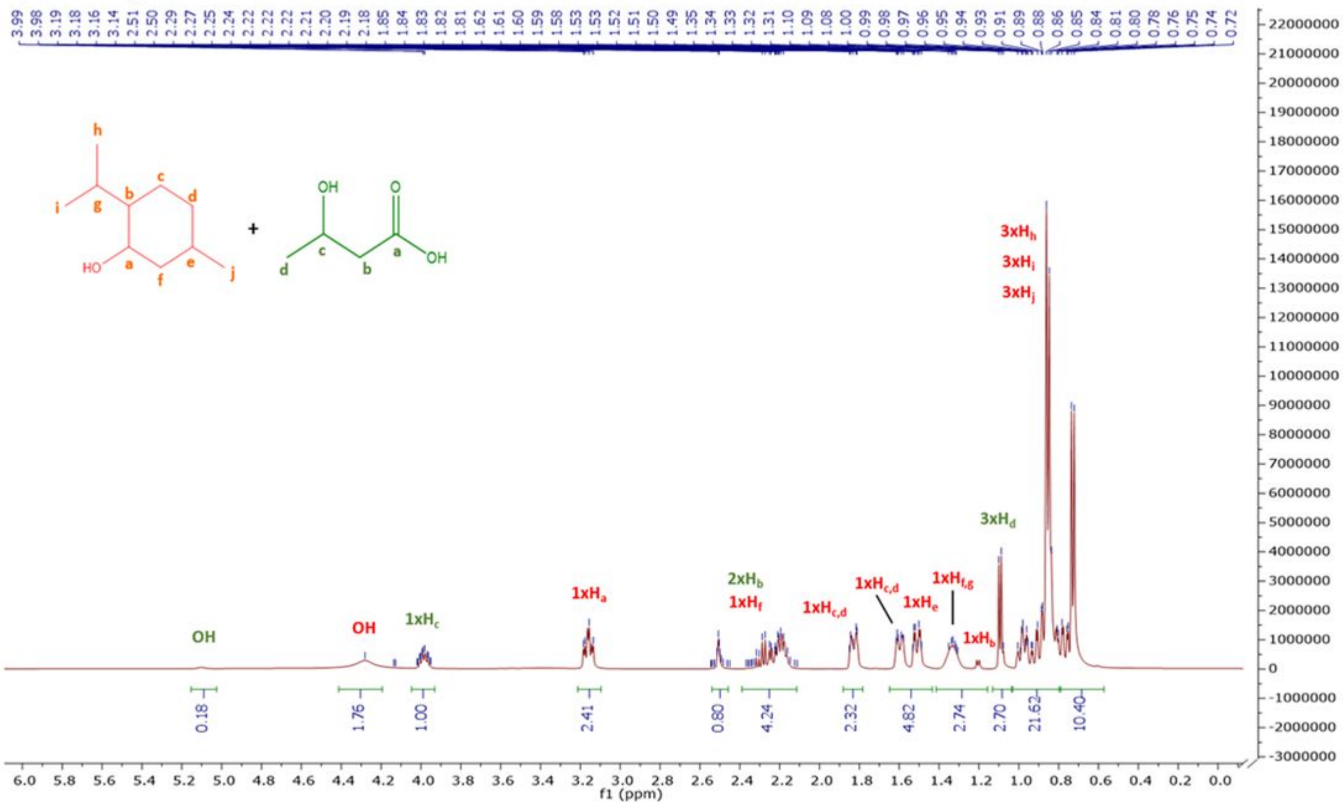

(c) Men:OL (1:1)

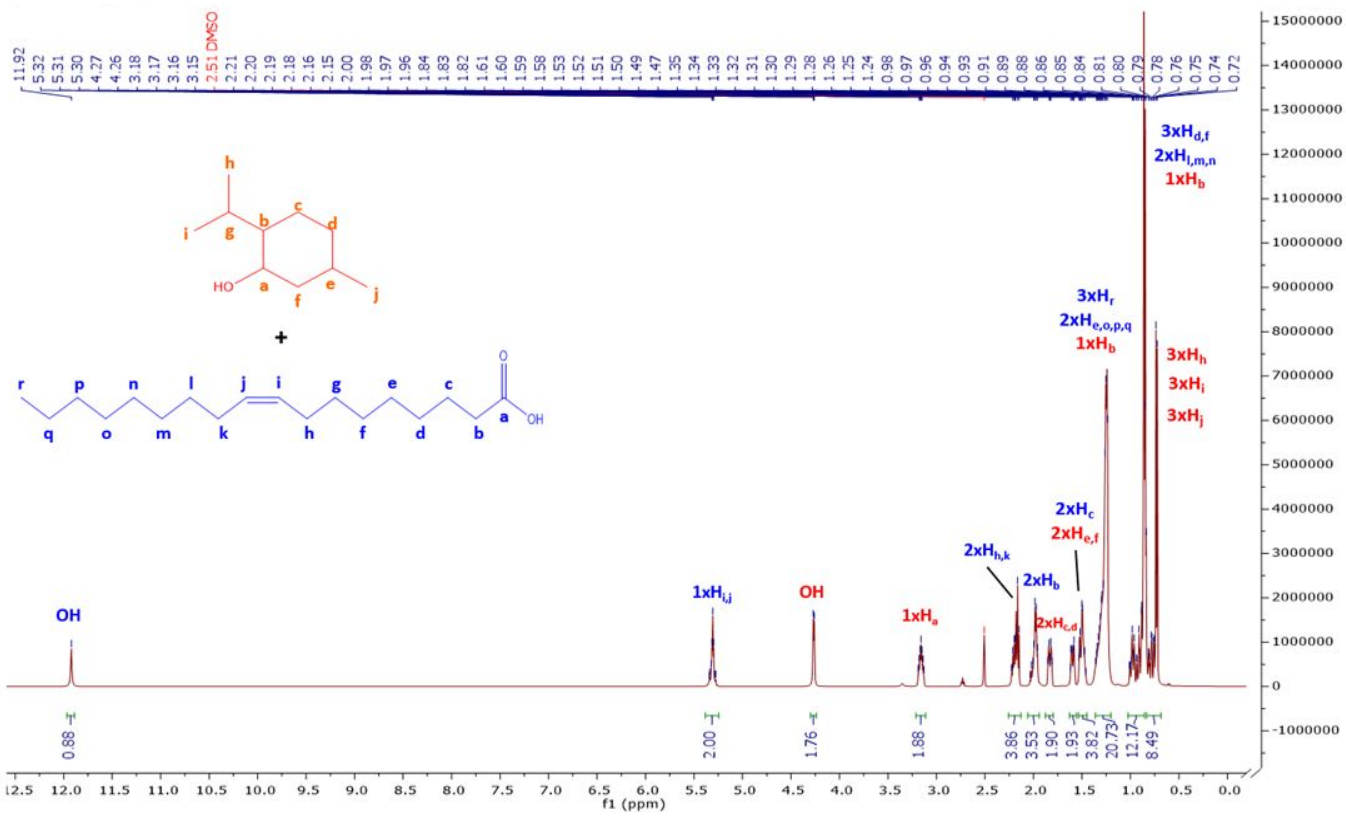

Figure S2. ^{1}H NMR spectra of HDES: (a) Men:HB (2:1), (b) Men:HB (3:1) e (c) Men:OL (1:1) in DMSO-d6.

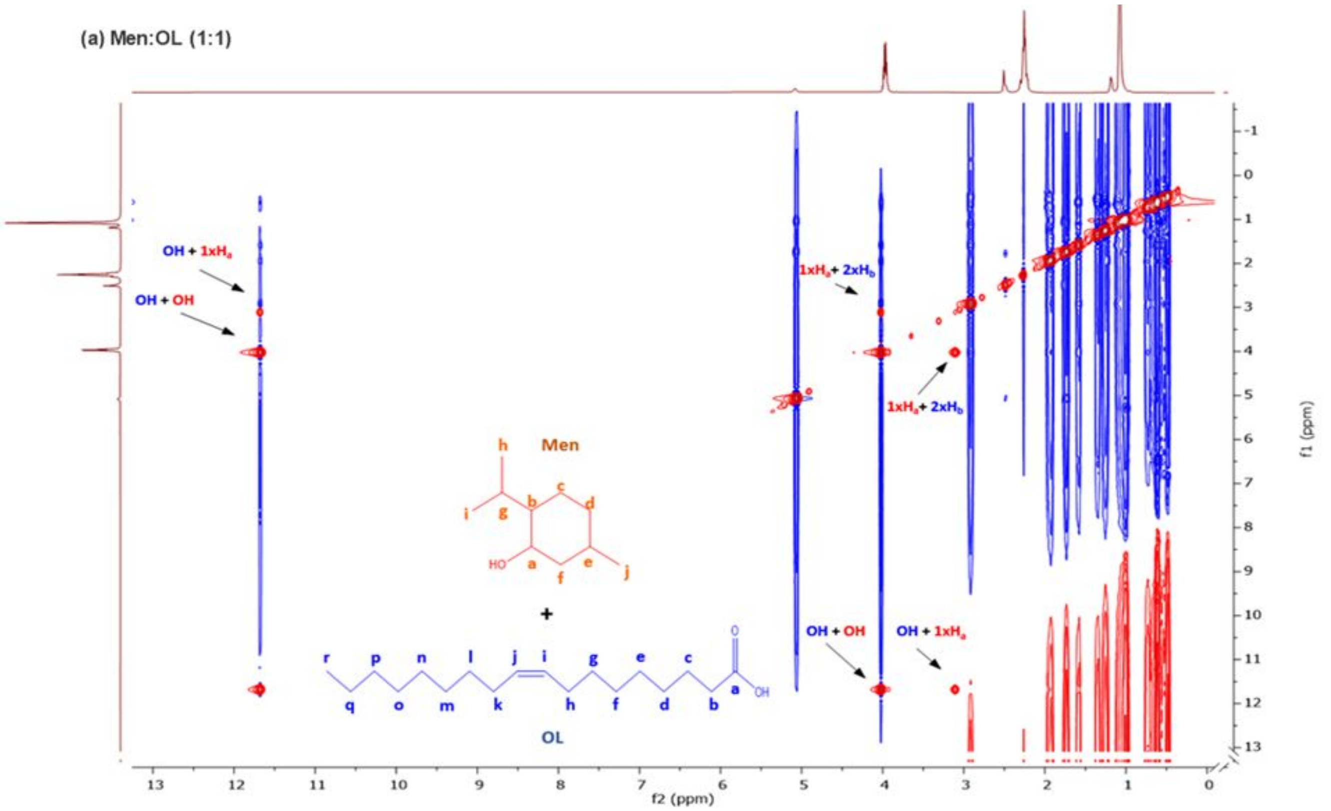
(a) Men:OL (1:1)
OH + 1xHa
OH + OH
1xHa+ 2xHb
1xHa+ 2xHb
OH + OH
OH + 1xHa
Men
OL
f2 (ppm)
f1 (ppm)

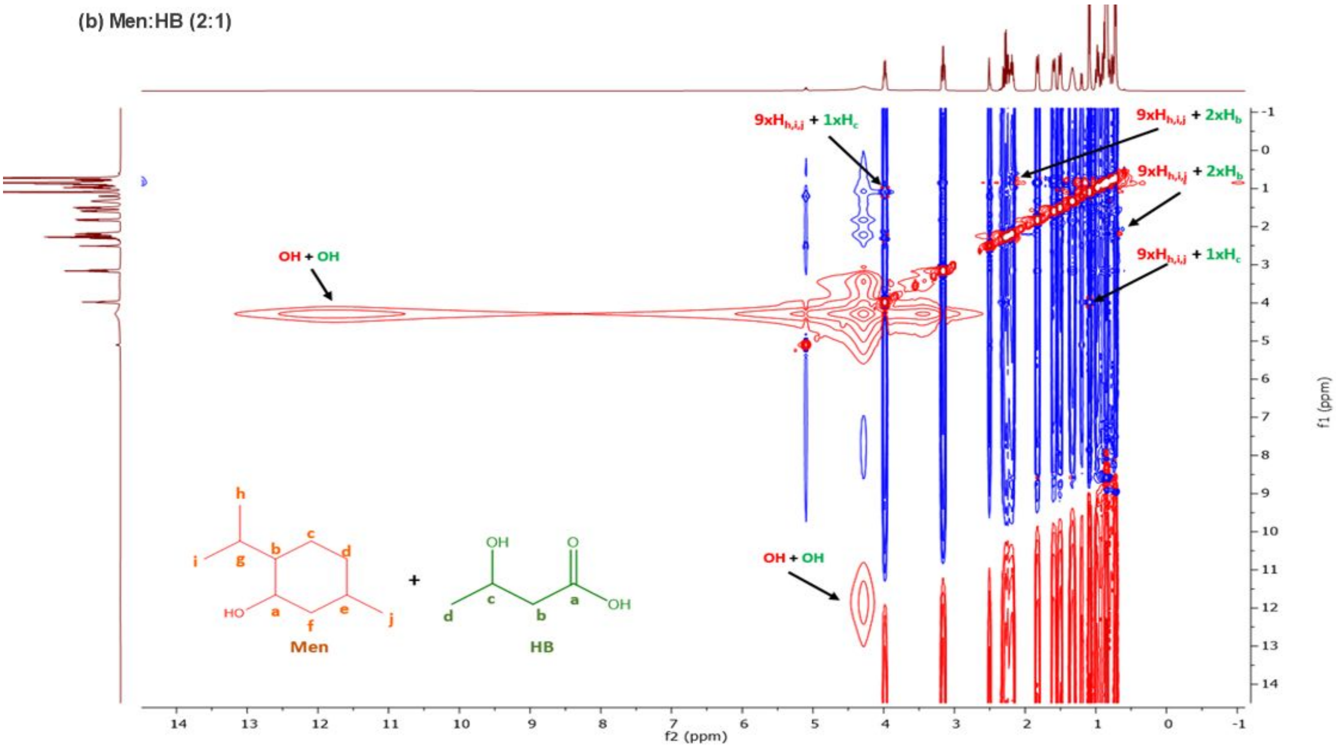
(b) Men:HB (2:1)
9xHh,i,j + 1xHc
9xHh,i,j + 2xHb
9xHh,i,j + 2xHb
9xHh,i,j + 1xHc
OH + OH
OH + OH
Men
HB
f2 (ppm)
f1 (ppm)

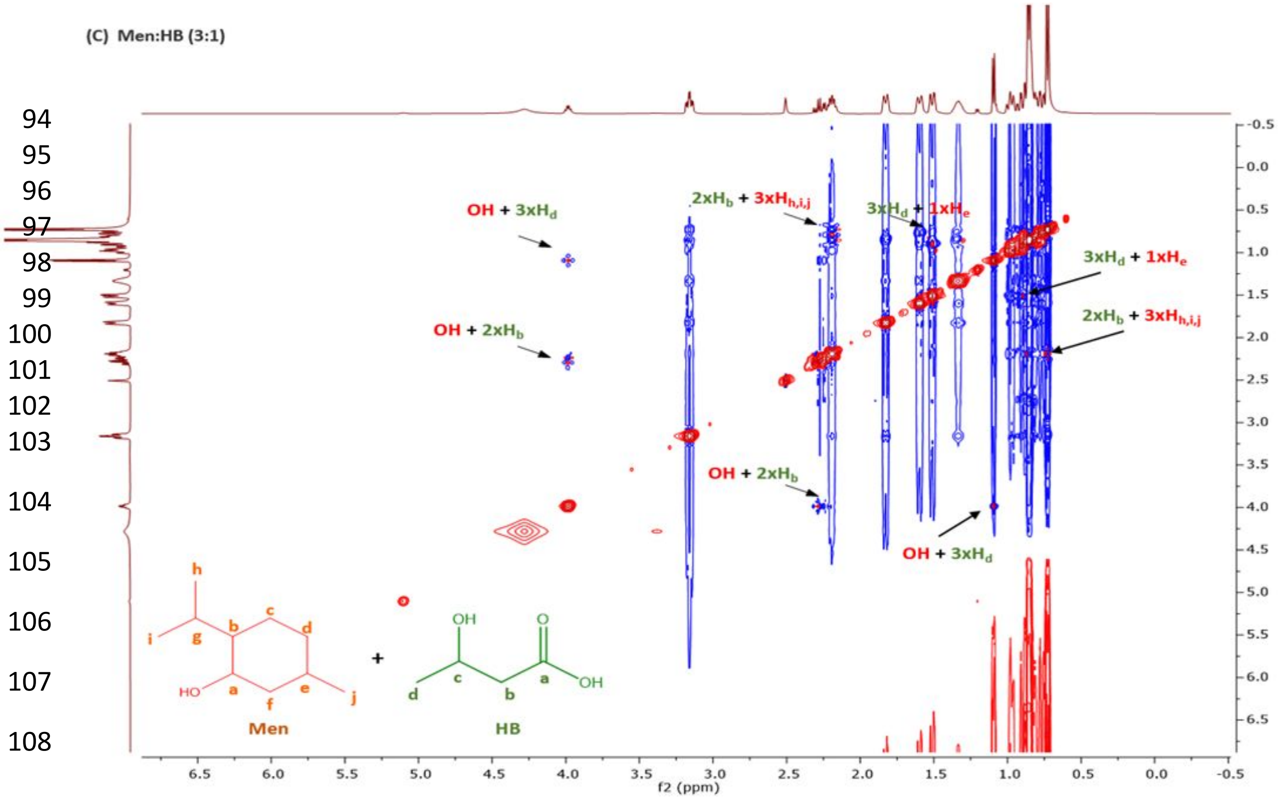

Figure S3. Two-dimensional NOESY spectra of HDES systems: (a) Men:OL (1:1), (b) Men:HB (2:1) and (c) Men:HB (3:1) in DMSO-d6..

Table S1. Biochemical assays performed with marine organisms. Significant differences recorded in the GST activity related to the assay of the incorporation of different concentrations of HDES in marine coating and simulation of its application in marine structures (painted plates), in mussels *Mytilus edulis* (gills (G) and digestive glands (D)) and limpets *Patella vulgata* (L). II – biocide-free coating (control); III – biocide-free coating + 2 mg/mL (HDES); IV - biocide-free coating + 5 mg/mL (HDES); V - biocide-free coating + 15 mg/mL (HDES); VI - biocide-free coating + 25 mg/mL (HDES); VII - biocide-free coating + 50 mg/mL (HDES); VIII - biocide-free coating + ivermectin.

		III			IV			V			VI			VII			VIII		
		G	D	L	G	D	L	G	D	L	G	D	L	G	D	L	G	D	L
II	G	---			---			---			---			---			---		
	D		p=0,0001			---			---			---			p=0,0119			---	
	L			---			---			---			---			---			---
III	G				p=0,0310			p=0,0257			---			---			p=0,0345		
	D					p=0,0001			p=0,0001			p=0,0001			p=0,0097			p=0,0001	
	L						---						---			---			
IV	G							---			---			---					
	D								---			---			p=0,0001				
	L									---			---			---			
V	G										---			---					

											---			p=0,0001					D
												---			---				L
VI													---						G
														p=0,0001					D
															---				L
VII																---			G
																	p=0,0002		D
																		---	L

Table S2. Biochemical assays in marine organisms. Significant differences recorded in the % SOD inhibition related to the assay of the incorporation of different concentrations of HDES in marine coating and simulation of its application in marine structures, in mussels *Mytilus edulis* (gills (G) and digestive glands (D)) and limpets *Patella vulgata* (L). II – biocide-free coating (control); III – biocide-free coating + 2 mg/mL (HDES); IV - biocide-free coating + 5 mg/mL (HDES); V - biocide-free coating + 15 mg/mL (HDES); VI - biocide-free coating + 25 mg/mL (HDES); VII - biocide-free coating + 50 mg/mL (HDES); VIII - biocide-free coating + ivermectin.

	III			IV			V			VI			VII			VIII			
	G	D	L	G	D	L	G	D	L	G	D	L	G	D	L	G	D	L	
II	---			---			---			---			---						G
		---			---			p=0,0471			---			---					D
			---			---			---			---			---				L
III				---			p=0,0377			---			---			---			G
					p=0,0496			p=0,0348			---			---			---		D
						---			---			---			---			---	L
IV							---			---			p=0,0464			---			G
								---			---			p=0,0148			---		D
									---			---			---			---	L
V										---			p=0,0020			---			G
											---			p=0,0100			---		D
												---			---			---	L
VI													p=0,0500			---			G
														---			---		D

															---			---	L
VII																---			G
																	---		D
																		---	L

Table S3. Biochemical assays in marine organisms. Significant differences recorded in lipidic peroxidation activity related to the assay of the incorporation of different concentrations of HDES in marine coating and simulation of its application in marine structures, in mussels *Mytilus edulis* (gills (G) and digestive glands (D)) and limpets *Patella vulgata* (L). II – biocide-free coating (control); III – biocide-free coating + 2 mg/mL (HDES); IV - biocide-free coating + 5 mg/mL (HDES); V - biocide-free coating + 15 mg/mL (HDES); VI - biocide-free coating + 25 mg/mL (HDES); VII - biocide-free coating + 50 mg/mL (HDES); VIII - biocide-free coating + ivermectin.

	III			IV			V			VI			VII			VIII			
	G	D	L	G	D	L	G	D	L	G	D	L	G	D	L	G	D	L	
II	---			---			---			---			---						G
II		---			---			---			---			---					D
II			---			---			---			---			---				L
III				---			---			---			---			---			G
III					p=0,0350			---			p=0,0052			---			---		D
III						---			---			---			---			---	L
IV							---			p=0,0304			---			---			G
IV								---			---			p=0,0371			---		D
IV									---			---			---			---	L
V										---			---			---			G
V											---			---			---		D
V												---			---			---	L
VI													---			---			G
VI														p=0,0040			---		D
VI															---			---	L
VII																---			G
VII																	---		D
VII																		---	L

Table S4. Biochemical assays in marine organisms. Significant differences recorded in the catalase activity related to the assay of the incorporation of different concentrations of HDES in marine coating and simulation of its application in marine structures, in mussels *Mytilus edulis* (gills (G) and digestive glands (D)) and limpets *Patella vulgata* (L). II – biocide-free coating (control); III – biocide-free coating + 2 mg/mL (HDES); IV - biocide-free coating + 5 mg/mL (HDES); V - biocide-free coating + 15 mg/mL (HDES); VI - biocide-free coating + 25 mg/mL (HDES); VII - biocide-free coating + 50 mg/mL (HDES); VIII - biocide-free coating + ivermectin.

	III			IV			V			VI			VII			VIII			
	G	D	L	G	D	L	G	D	L	G	D	L	G	D	L	G	D	L	
II	---			---			---			---			---						G
		---			---			---			p=0,0424			---					D
			---			---			---			---			---				L
III				---			p=0,0447			---			---			---			G
					---			p=0,0170			p=0,0002			---			---		D
						---			---			---			---			---	L
IV							---			---			---			---			G
								---			---			---			---		D
									---			---			---			---	L
V										---			p=0,0071			---			G
											---			p=0,0067			---		D
												---			---			---	L
VI													p=0,0424			---			G
														p=0,0001			---		D
															---			---	L
VII																p=0,0040			G
																	---		D
																		---	L
